# High‐impedance alerts with pulse generator—Lead mismatch

**DOI:** 10.1002/joa3.12745

**Published:** 2022-06-14

**Authors:** Simon Christie, Nada El Tobgy, Colette M. Seifer, Clarence Khoo

**Affiliations:** ^1^ Max Rady College of Medicine, Faculty of Health Sciences University of Manitoba Winnipeg Canada

**Keywords:** high‐impedance alert, pacemaker, pacemaker Lead mismatch

## Abstract

**Background:**

Cardiac Implantable Electronic Devices (CIED) include pulse generators and leads. In some implanting centers, it is a common practice to combine devices with leads from different companies. Case series have reported episodic high‐impedance changes in Boston Scientific CIEDs with competitor leads. We investigated the incidence of high‐impedance abnormalities in matched versus mismatched Boston Scientific Accolade pacemakers.

**Methods:**

A retrospective chart review identified all consecutive Boston Scientific Accolade pacemakers implanted between January 2017 and June 2019 at a Canadian tertiary care center. The primary outcome was the occurrence of transient, high‐impedance changes which resulted in a switch to unipolar pacing/sensing in the absence of any other identifiable lead issue. Fisher exact tests (two‐tailed, *α* = .05) were used to compare the incidence of outcomes in matched versus mismatched systems.

**Results:**

514 Boston Scientific Accolade pacemakers were associated with 882 individual leads. The primary outcome occurred with 21 leads (20 Medtronic and 1 Abbott), associated with occasional pacing inhibition, presyncope, and/or early surgical revision. Mismatched lead‐device pairs were significantly associated with CIED malfunction compared to matched lead‐device pairs (3.3% vs. 0%, *p* = .0019). The median time from implant to unipolar safety switch was 12.4 months. The median follow‐up time was 21.6 months.

**Conclusion:**

Use of mismatched leads with a Boston Scientific Accolade device was associated with an increased risk of undesirable changes in sensing polarity with occasional inappropriate pacing inhibition. Awareness of this interaction can allow for the institution of appropriate programming remedies and may increase scrutiny of the use of mismatched CIED systems.

## Graphical abstract

Use of mismatched leads with a Boston Scientific Accolade device was associated with increased risk of undesirable changes in sensing polarity and adverse clinical outcomes. Awareness of this interaction can allow for institution of appropriate programming remedies and may increase scrutiny of the use of mismatched CIED systems.

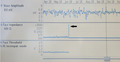

## 1 INTRODUCTION

Cardiac implantable electronic devices (CIED), including pacemakers and implantable cardioverter defibrillators (ICD), are comprised of a pulse generator as well as leads produced by several manufacturers. In some high‐volume implanting centers, it is common practice to mix and match devices with leads from different companies. This may be the result of operator preference for certain leads or because of quotas set forth by hospital‐vendor contracts. Recently, case series have reported transient, episodic high‐impedance alerts in Boston Scientific CIEDs when implanted with competitor leads which can result in lead noise and pacing suppression.^1^, ^2^, ^3^, ^4^ Our study sought to investigate the incidence of spontaneous high‐impedance events in matched versus mismatched Boston Scientific CIED systems.

## 2 MATERIAL AND METHODS

A retrospective chart review identified all consecutive patients implanted with a Boston Scientific Accolade pacemaker between January 2017 and June 2019 at a Canadian tertiary care cardiac center. Data extracted included indication for implantation, device model, lead(s) model, date of implantation, and any documented lead noise or abnormalities in lead testing. Follow‐up data were collected until December 2020. The study was approved by our institutional research ethics board.

The primary outcome was the occurrence of transient, high‐impedance lead measurements resulting in a safety switch to unipolar pacing/sensing in the absence of any other identifiable lead issue. Provocative maneuvers were performed in the clinic, including pocket manipulation and isometric exercises to attempt to identify an underlying lead or insulation fracture. Other lead outcomes not related to the primary outcome were also tracked.

All patients were scheduled for regular follow‐up in the Pacemaker Clinic at 1, 6, and 12 months post‐implantation, followed by annual visits thereafter. Patients were seen outside of these predetermined intervals if any patient or CIED concerns arose.

During the study period, an advisory was circulated regarding intermittent oversensing of the minute ventilation (MV) sensor in certain Boston Scientific CIEDs.^5^ As per the recommendations, the MV sensor was turned off in all subsequent new implants. All patients with prior implants were recalled to the clinic to have the MV sensor turned off.

### 2.1 Statistics

A matched lead‐device pair was defined as a Boston Scientific lead paired with the Boston Scientific Accolade device, whereas a mismatched lead‐device pair was defined as a lead (either atrial or ventricular) from a competitor company paired with the Accolade device.

Given that the variable under investigation was a lead‐device mismatch, all statistical analyses were performed on each individual lead‐device pair. While single‐chamber pacemakers would provide a single lead‐device pair, dual‐chamber pacemakers would provide two lead‐device pairs (one corresponding to the atrial lead and one to the ventricular lead). As a result, a dual‐chamber pacemaker might contribute two matched lead‐device pairs, two unmatched lead‐device pairs, or one of each (e.g., Boston Scientific atrial lead and Medtronic ventricular lead).

Data are provided as median ± interquartile range unless otherwise specified. Comparison of occurrence of the primary outcome between matched and mismatched lead‐device pairs was performed using Chi‐square tests or Fisher Exact tests, as appropriate. All comparisons were two‐tailed, with an *α* = .05. All statistical analyses were performed using SPSS Version 27.0 (IBM Corp., Armonk, NY, USA).

## 3 RESULTS

### 3.1 Patient and CIED system characteristics

**FIGURE 1:**
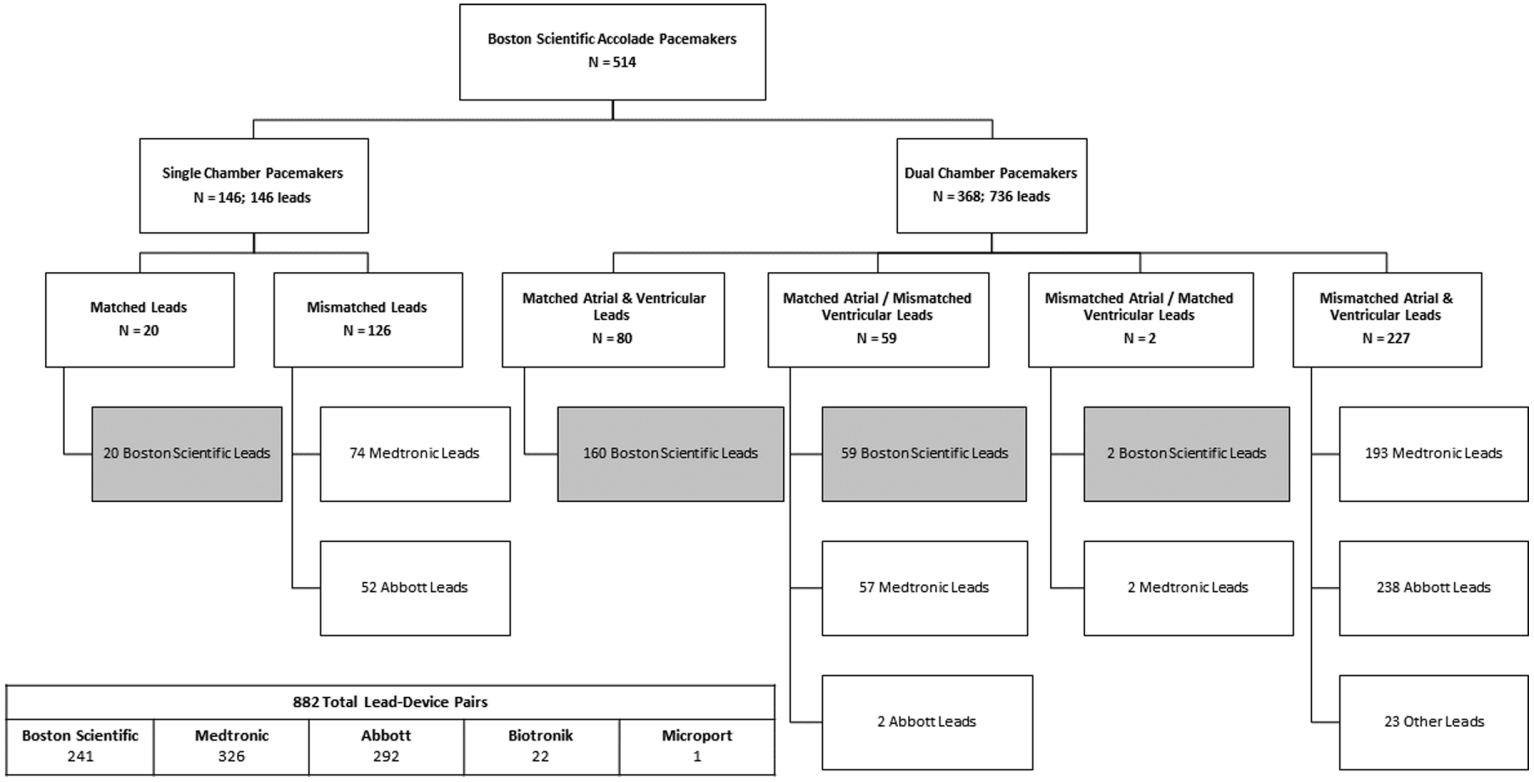
Distribution of matched and mismatched lead‐device pairs within the study population. “N” values denote the number of patients with specified pacing systems; all other values denote the number of lead‐device pairs. Boxes with grey background denote matched lead‐device pairs.

A total of 514 patients implanted with an Accolade device were identified. These pulse generators were associated with 882 total leads, including 241 Boston Scientific matched lead‐device pairs, and 641 competitors mismatched lead‐device pairs including 326 Medtronic and 292 Abbott leads (Figure 1). A total of 436 patients received a de novo CIED system, and 78 patients underwent replacement of their CIED pulse generator. There were 368 dual‐chamber devices and 146 single‐chamber devices implanted. The majority of systems included one or more mismatched lead‐device pairs. Only 100/514 (19.5%) pacemakers were associated with completely matched lead‐device pairs, while 353/514 had completely mismatched lead‐device pairs (68.6%); the remaining 61/514 (11.9%) had a combination of matched and mismatched lead‐device pairs. There were a total of 241/882 (27.3%) matched lead‐device pairs, and 641/882 (72.7%) mismatched lead‐device pairs. The predominant indication for implantation was AV node dysfunction/heart block (72.8%). Twenty‐seven patients (5.2%) either died or were lost to follow‐up without an initial clinic visit after implantation. Baseline characteristics of all patients and their CIED systems are shown in Table 1.

**TABLE 1:** Baseline characteristics of study patients

Characteristic	Study population (*N* = 514)
Age	
Median (IQR)	80 (71–87)
Female sex (%)	41.6
Indication for pacing	
AV Node dysfunction	374
Sick sinus syndrome	85
Tachy‐Brady syndrome	43
Syncope / Rhythm not documented	12
Dual / Single Chamber Pacemaker	368/146
New systems (%)	85

### 3.2 Primary outcome

Twenty‐one individual leads (11 atrial leads and 10 ventricular leads) developed transient, unexplained high‐impedance alerts causing a polarity switch among 19 separate patients, 20 of which occurred with Medtronic leads (20/326, 6.1%), and 1 with Abbott (1/292, 0.3%). Two patients had a safety switch occur on both the atrial and ventricular leads on separate occasions. No episodes of unexplained high‐impedance alerts were noted among matched systems. The primary outcome in mismatched lead‐device pairs occurred at a rate of 3.3% (21/641) versus 0% (0/241) in matched lead‐device pairs (*p* = .0019). The use of Medtronic leads was significantly associated with the primary outcome compared to matched lead‐device pairs (*p* ≤ .00001) whereas those using Abbott leads were not statistically significant (*p* = 1). The median time from implantation to the first documented abnormality in the clinic was 19.3 months (IQR 10.6–29.5). The median time to safety switch trigger was 12.4 months (IQR 8.8–19.1). The median follow‐up time was 21.6 months (IQR 13–31).

**FIGURE 2:**
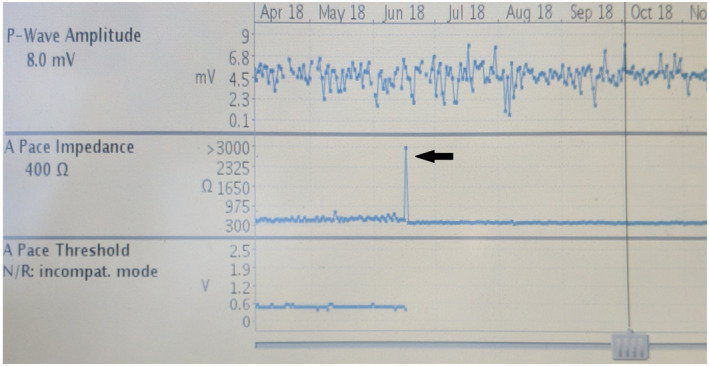
Lead impedance trends of an atrial lead with safety switch obtained during pacemaker interrogation. Note the sudden and transient spike in atrial lead impedance to >3000 Ω (arrow) with stable, normal lead impedances before and after. In this patient, an atrial lead safety switch was triggered in association with the lead impedance spike.

A review of impedance trends revealed that these high‐impedance events were all single, isolated measurements with all other impedance measurements within a normal range (Figure 2). All events were identified during in‐clinic visits as remote monitoring for pacemakers was not standard‐of‐care at our site during the follow‐up period. Fourteen patients had imaging performed after the discovery of the issue with no gross lead fracture or pin‐header connection issues identified. Provocative maneuvers performed in the clinic, including pocket manipulation and isometric exercises, did not reproduce the abnormalities in any of the 20 patients. Of note, all events occurred after the MV sensor had already been turned off. All cases are shown in Table 2. Nineteen high‐impedance alerts occurred with new device implants, whereas 2 occurred in patients following routine pulse generator replacement for end of battery life.

**TABLE 2:** Characteristics of individuals with primary outcome

Age	Sex	Pulse generator	Ventricular lead	Atrial lead	Indication for pacing	Significant adverse outcomes
70	F	L311 MRI	Med 5076 CapsureFix Novus	Med 5076 CapsureFix Novus^a^	AVN dysfunction	
78	M	L310 MRI	Med 5076 CapsureFix Novus^a^		AVN dysfunction	
79	M	L310 MRI	Med 5076 CapsureFix Novus^a^		AVN dysfunction	Presyncope resulting in Surgical Device Revision
87	F	L311 MRI	Med 4076 Capsure Sense	Med 4076 Capsure Sense^a^	AVN dysfunction	
68	F	L331 ‐ MRI EL	Med 4076 Capsure Sense^a^	Med 4076 Capsure Sense	SSS	Presyncope
69	M	L310 MRI	Med 4076 Capsure Sense^a^		AVN dysfunction	
78	F	L331 ‐ MRI EL	Med 4076 Capsure Sense	Med 4076 Capsure Sense^a^	SSS	
80	M	L331 ‐ MRI EL	Med 4076 Capsure Sense	Med 4076 Capsure Sense^a^	AVN dysfunction	
81	M	L331 ‐ MRI EL	Med 4076 Capsure Sense	Med 4076 Capsure Sense^a^	AVN dysfunction	
78	F	L331 ‐ MRI EL	Med 4076 Capsure Sense^a^	Med 4076 Capsure Sense^a^	AVN dysfunction	Syncope
74	F	L331 ‐ MRI EL	Med 4076 Capsure Sense^a^	Med 4076 Capsure Sense^a^	AVN dysfunction	Presyncope resulting in Surgical Device Revision
60	F	L311 MRI	Med 4076 Capsure Sense	Med 4076 Capsure Sense^a^	AVN dysfunction	Presyncope
77	M	L310 MRI	Med 5076 CapsureFix Novus^a^		AVN dysfunction	Syncope
79	F	L311 MRI	Med 4076 Capsure Sense	Med 4076 Capsure Sense^a^	Tachy‐Brady	Presyncope, falls
86	M	L310 MRI	Med 4076 Capsure Sense^a^		SSS	
59	M	L331 MRI EL	Med 4076 Capsure Sense	Med 4076 Capsure Sense^a^	AVN dysfunction	
70	F	L331 MRI EL	Med 4076 Capsure Sense^a^	Med 4076 Capsure Sense	AVN dysfunction	
72	F	L311 MRI	Abbott 1688TC Tendril SDX	Abbott 1688TC Tendril SDX^a^	AVN dysfunction	
27	F	L331 MRI EL	Med 5076 Capsure Sense^a^	Med 5076 Capsure Sense	AVN dysfunction	

Abbreviations: AVN, Atrioventricular Node; Med, Medtronic; SSS, Sick Sinus Syndrome.

^a^ Lead associated with primary outcome.

**FIGURE 3:**
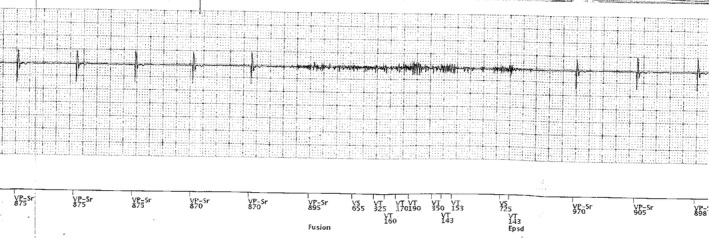
Intracardiac tracing demonstrating inappropriate ventricular pacing inhibition because of oversensing of lead noise in unipolar sensing mode. Note artifactual noise on the ventricular channel with the absence of ventricular pacing or intrinsic ventricular activity. This particular event corresponded to patient complaints of presyncope. VP = ventricular paced event, VS = ventricular sensed event, VT = ventricular sensed event annotated as ventricular tachycardia because of short V‐V interval.

Two patients reported syncopal events after the safety switch occurred on their ventricular leads, but there was no documented arrhythmia on device interrogation to show causation. However, as ventricular pacing inhibition caused by intermittent, transient lead noise may not consistently trigger a recorded arrhythmia event, this must be still considered a possible etiology for syncope. Multiple additional patients reported significant presyncopal symptoms including 2 patients with documented episodes of prolonged pacing inhibition on the ventricular lead (some episodes >1 min) secondary to lead noise. One representative example of ventricular lead noise resulting in presyncope is shown in Figure 3.

### 3.3 Management of primary outcome

Early during the time period described in this study, there was a lack of appreciation of the mechanism behind the sporadic safety switches that were observed and consequently, no consistent management strategy was followed. Occurrence of the safety switch was initially managed with the reprogramming of the device back to bipolar sense and bipolar pace. Five patients reprogrammed to this configuration had more than one safety switch occur repeatedly on the same lead. These repeat episodes were not counted as new primary outcome events.

During this early phase, 2 patients with significant pauses secondary to inappropriate pacing inhibition underwent early surgical device revision following consultation with Boston Scientific technical support. This decision was driven by the severity of their clinical presentation and their inability to reliably reprogram their device back to consistent bipolar sensing. In these 2 cases, given that the leads tested normally through the pacing system analyzer during the revision procedure, it was surmised that the problem may have been with the pulse generator header and their pulse generators were therefore replaced with a matched device. No further primary outcomes were observed after the change to a matched pulse generator.

Following recognition by Boston Scientific that this may be a systemic issue, technical support recommended that patients identified with the primary outcome have their device reprogrammed to unipolar pacing with bipolar sensing. All patients with mismatched lead‐device pairs still followed by the clinic were subsequently reprogrammed to these settings. No further episodes of inappropriate safety switches have been documented after reprogramming to unipolar pacing with bipolar sensing.

### 3.4 Other Lead outcomes

Fifteen additional lead issues were identified in the entire cohort including 6 chronically high thresholds, 3 unexplained noise on lead, 2 noise on lead with MV sensor still on, 2 noise on lead reproducible with pocket manipulation, 1 lead dislodgement, and 1 loss of capture. These other lead outcomes occurred with 2 Boston Scientific leads and 13 other leads. There was no statistical difference between matched versus mismatched lead‐device pairs among these other lead outcomes (*p* = .38).

## 4 DISCUSSION

Despite all available leads and headers meeting international regulatory requirements, there have been reports in the literature documenting a higher rate of adverse device outcomes with mismatched systems. Case series have documented transient high‐impedance events in patients with a combination of a Boston Scientific ICD and either Medtronic or Abbott leads.^1^, ^2^, ^4^ Recently, a large study of “hybrid” pacing systems incorporating Boston Scientific pacemakers and mismatched leads demonstrated a 9% rate of transient impedance increases.^3^ To our best knowledge, our study is the largest series of Boston Scientific pulse generators with mismatched leads in the literature and confirms that there is a significant increase in the occurrence of transient impedance increases with mismatched lead‐device pairs compared to matched lead‐device pairs.

The incidence of the primary outcome in our series is numerically lower than that reported in Karnik et al. (3.3% vs. 9%).^3^ Part of this difference may be the result of differences in the proportion of lead manufacturers used between the two studies. Our study included a much higher number of Medtronic leads compared to Karnik et al. (326 vs. 18) and will therefore more accurately reflect the true incidence of the primary outcome with this lead‐pulse generator combination. However, Karnik et al. reported a much higher proportion of lead fracture among Abbott leads compared with our study. This difference may be the result of different leads used—whereas their study used the Abbott Tendril 1688TC, 1388TC, and 1888ST, our study used a large proportion of Abbott Tendril 2088TC. In fact, our only primary outcome with an Abbott lead occurred with a 1688TC.

Potential mechanisms for the primary outcome have been put forth in the Boston Scientific Minute Ventilation (MV) advisory as well as by Tannawuttiwat et al.^2^, ^5^ The MV sensor advisory was released in December 2017 and outlined reports of intermittent oversensing of the MV sensor signal with certain Boston Scientific CIEDs. It was noted that the use of different surface finishes in the lead terminal ring electrode and pin‐header interface design resulted in subtle differences in axial and radial motion that may allow for micro‐abrasions to occur causing particulate matter to accumulate. In addition, differences exist in the spring contacts used to interface between the header and the pin, both in the structure of the spring and in the contact force exerted by the spring on the pin. These differences may also influence the chance for micro‐abrasions to occur.^2^ These particles may oxidize and temporarily interfere with contact between the pin and header, thus triggering high‐impedance changes. Depending on the context, these high‐impedance changes may result in both oversensing of MV sensor signals as seen in the MV sensor advisory or result in a safety switch change as seen in the primary outcome of our study.^2^, ^5^ The risk of device malfunction with the MV sensory was significantly greater when competitor leads were used with the Boston Scientific pulse generator, with a 1 in 2000 probability of injury in 5 years for Medtronic or Abbott pacing leads compared to 1 in 33 333 for matched leads.^5^ Our study confirms that this increased risk of lead‐device issues with mismatched competitor leads also extends to the sudden safety switch changes encountered with our primary outcome. While a software solution called the Signal Artifact Monitor (SAM) has been created to eliminate the clinical risk of pacing inhibition caused by the MV sensor issue, this software update did not address the primary outcome identified by our study, as in all cases the MV sensor was turned off prior to the occurrence of the safety switch.

Once a high‐impedance alert occurs, this triggers a safety switch which causes the device to be reprogrammed to unipolar pacing and sensing, thus increasing the potential of oversensing and inappropriate pacing inhibition. A manufacturer‐proposed programming solution to this issue is to change the device to unipolar pacing with bipolar sensing. It is hypothesized that the ring electrode is more susceptible to the above issues with micro‐abrasions. By programming the lead to unipolar pacing, the ring electrode is excluded from impedance testing, thus avoiding high‐impedance readings and reducing the risk of a safety switch being triggered. Since implementing this programing solution in our patients, we have not identified any further recurrence of an inappropriate safety switch.

A solution employed for some patients in our study was to surgically revise their device such that the pulse generator matched the existing leads. While the number of patients in our study who underwent surgical revision was small, the elimination of the primary outcome in all these patients lends further credence to the mismatch itself being the cause of the primary outcome, and not the leads themselves. The surgical revision comes with significantly increased risk to the patient and thus programming changes should be first employed. However, surgical revision may still be necessary for patients with repeated high‐impedance alert episodes despite programming changes, or where there is a concern of a high chance of clinical compromise with another event.

While our study highlights a specific interaction occurring with a subset of Boston Scientific pulse generators and mismatched leads, there are other reasons why mismatched “hybrid” systems may not be ideal. One key consideration is that while MRI‐conditional components are provided by most manufacturers, the CIED system is only considered to be MRI‐conditional in “matched” systems with all components provided by the same manufacturer.^6^ In addition, in the event of a major lead advisory, attempts at risk mitigation by the lead manufacturer have historically been through detection algorithms or remote monitoring systems built into their pulse generators (e.g., Medtronic Sprint Fidelis advisory and Medtronic Lead Integrity Alert), which discourages against the use of mismatched lead‐device pairs.

There are multiple reasons why mixing and matching leads with pulse generators from competing companies occurs. Implanting physicians may choose to mix and match leads and pulse generators based on lead handling characteristics and overall familiarity with particular models. Hospitals and health care regions may also commit to bulk purchasing contracts where the most cost‐efficient lead and pulse generators are provided by different manufacturers. As noted above, the use of these mismatched systems may result in a number of adverse outcomes for the patient, including potentially unexpected lead‐device interactions as outlined in our study.

There are scenarios where mixing and matching may be unavoidable, such as the replacement of existing devices at the end of battery life where the original lead company no longer exists. If a mismatched system is implanted, it may be prudent to schedule more frequent follow‐ups and patients should be made aware that they may be at increased risk of unanticipated lead‐pulse generator interactions.

## 5 CONCLUSION

Among patients with a Boston Scientific Accolade device, the use of mismatched leads was associated with a significant increase in lead impedance alerts with a subsequent safety switch to unipolar pacing and sensing with occasional inappropriate pacing inhibition. Awareness of this interaction can allow for the institution of appropriate programming remedies and may increase scrutiny of the use of mismatched CIED systems. The results of this study suggest that matched lead‐pulse generator systems be preferred over mismatched systems.

## CONFLICT OF INTEREST

None.

## Data Availability

The data underlying this article will be shared upon reasonable request to the corresponding author.
